# Overcoming Challenges in Cancer Care: A Focus on Emerging Technologies

**DOI:** 10.3390/biomedicines12122787

**Published:** 2024-12-09

**Authors:** Aya Hasan Alshammari, Takaaki Hirotsu, Eric di Luccio

**Affiliations:** Hirotsu Bioscience Inc., New Otani Garden Court 22F, 4-1 Kioi-cho, Chiyoda-ku, Tokyo 102-0094, Japan; hirotsu@hbio.jp (T.H.); e.diluccio@hbio.jp (E.d.L.)

## 1. Introduction

Cancer research is rapidly evolving, propelled by advancements in molecular biology, genomics, and immunology. This Special Issue, “Advanced Cancer Diagnosis and Treatment”, highlights cutting-edge research addressing critical oncology challenges, including early detection, therapeutic resistance, and personalized medicine.

This editorial synthesizes critical findings and explores future directions in therapeutic innovation, diagnostic advancements, immune modulation, and precision oncology. By fostering a deeper understanding of cancer biology and clinical applications, we aim to improve patient outcomes and advance global cancer care.

## 2. Advancing Therapeutics: Exploring Novel Frontiers

The field of oncology is undergoing a paradigm shift driven by innovative therapeutic strategies aimed at overcoming treatment resistance, exploiting tumor-specific vulnerabilities, and improving patient outcomes. This Special Issue highlights several cutting-edge approaches that exemplify these advancements while addressing critical challenges in their clinical translation.

One promising avenue is the development of miRNA-based therapies for gastrointestinal cancers. As detailed in the review article “Drug Discovery and Development of miRNA-Based Nucleotide Drugs for Gastrointestinal Cancer”, miRNAs such as miR-302 and miR-451 have demonstrated significant potential in regulating oncogenic pathways, reprogramming epigenetic mechanisms, and enhancing chemotherapy sensitivity. Beyond their therapeutic potential, miRNAs also serve as dynamic, non-invasive biomarkers, offering real-time insights into tumor progression and treatment response. However, the journey from bench to bedside is far from straightforward. While advances in delivery systems, such as lipid nanoparticles and extracellular vesicles, have improved miRNA stability and specificity, challenges persist. Off-target effects, variability in patient response, and the degradation of miRNAs in vivo remain significant barriers. Moreover, the cost and complexity of these technologies may limit their accessibility, particularly in low-resource settings [1].

To unlock the potential of miRNA therapies, investment in cost-effective delivery systems, such as biosimilars, is crucial. Translational research must also be expanded through large-scale trials in diverse populations to validate efficacy and safety. Additionally, integrating AI and computational biology could refine target selection, predict resistance mechanisms, and accelerate the development of personalized treatment regimens. These strategies are essential to bridging the gap between innovation and real-world clinical application [1,2].

Targeting metabolic vulnerabilities presents another groundbreaking approach. In hepatocellular carcinoma (HCC), SMARCA4 inhibition has demonstrated the ability to induce ferroptosis, a form of cell death that selectively targets tumor cells by disrupting lipid peroxidation. However, the metabolic adaptability of tumors often undermines sustained efficacy. Combining SMARCA4 inhibitors with complementary treatments, such as GPX4 inhibitors, could enhance therapeutic outcomes by disrupting compensatory pathways. Computational tools for mapping metabolic pathways offer further opportunities to identify vulnerabilities and refine combination strategies, broadening the applicability of this approach to other malignancies, such as KRAS-mutant pancreatic cancer [3].

Nanotechnology represents a third transformative frontier. Innovations like gold nanocages functionalized with anti-CD133 antibodies are redefining treatment paradigms for aggressive cancers like glioblastoma. These nanostructures combine therapeutic and diagnostic functions, enabling real-time monitoring alongside targeted drug delivery. While preclinical studies show significant reductions in glioblastoma stem-like cell viability, issues such as scalability, immunogenicity, and regulatory barriers hinder clinical adoption. Exploring combination strategies, such as pairing nanocages with immune checkpoint inhibitors or photothermal therapy, could accelerate translation and improve outcomes [4].

Immunotherapy combinations, such as the dual-action regimen of camrelizumab and lenvatinib for advanced gallbladder cancer, further exemplify the potential of innovative therapies. By simultaneously targeting tumor angiogenesis and reinvigorating the immune system, this approach has demonstrated improved progression-free survival compared to traditional chemotherapy. However, ensuring accessibility remains a challenge. Developing biosimilars, streamlining production pipelines, and refining predictive biomarkers like PD-L1 expression are critical to reducing costs and increasing global availability. Policy reforms, including subsidies, reimbursement programs, and international collaborations, are essential to bridging socioeconomic and geographic disparities [5].

These advancements collectively illustrate the persistent progress in oncology, driven by molecular precision, innovative delivery systems, and a commitment to improving accessibility. However, critical gaps remain. Translational research must address resistance mechanisms and validate these therapies in diverse populations. Ethical considerations, such as ensuring equitable access and affordability, cannot be overlooked. Overcoming these challenges will require interdisciplinary collaboration, global health policy reforms, and a sustained focus on patient-centered solutions.

This pivotal moment demands immediate action—one that calls for bold, coordinated action to ensure that these transformative therapies reach their full potential to improve patient care worldwide.

## 3. Diagnostic Innovations: Enhancing Early Detection and Precision

Early cancer detection is a cornerstone of improving survival rates [6], yet many current diagnostic tools fall short, particularly for asymptomatic or hard-to-detect malignancies [7]. Traditional methods like mammography struggle with dense breast tissue [8], while aggressive cancers like pancreatic and glioblastoma are often detected only at advanced stages [9,10]. This underscores an urgent need for innovative, sensitive, and accessible diagnostic solutions that adapt to diverse healthcare environments.

Innovations like the N-NOSE urine test and volatile organic compound (VOC) profiling are transforming pancreatic cancer detection. The N-NOSE test employs the extraordinary olfactory sensitivity of nematodes to identify cancer-specific chemical signatures in urine. At the same time, VOC profiling detects unique metabolic byproducts associated with cancer in bodily fluids [11].

For breast cancer, tools like Automated Breast Ultrasound (ABUS) and Contrast-Enhanced Mammography (CEM) are addressing the limitations of traditional mammography, particularly in women with dense breast tissue. ABUS, a non-ionizing imaging technology, demonstrates a sensitivity of 72% in dense breast cases compared to 29% for conventional mammography. Similarly, CEM enhances vascular imaging, allowing better characterization of ambiguous lesions and improving detection rates [12].

While these advancements significantly enhance diagnostic accuracy, barriers such as cost, accessibility, and the need for specialized training hinder widespread adoption [13]. Potential solutions include subsidized diagnostic programs for underserved populations, mobile imaging units for remote areas, and streamlined imaging protocols [14,15]. Integrating AI into imaging workflows could amplify the impact of these technologies by automating lesion detection, prioritizing high-risk cases, and reducing the burden on radiologists [16].

The urgency for better early detection cannot be overstated. These innovations have the potential to save countless lives, but their true impact depends on overcoming barriers to accessibility and equity. By addressing these challenges, we can ensure that advanced diagnostics reach all patients, transforming cancer care and improving outcomes worldwide.

## 4. Immune Modulation: Shaping the Future of Cancer Therapy

Harnessing the immune system to combat cancer has emerged as a cornerstone of modern oncology, presenting unprecedented opportunities to address therapeutic resistance, tumor immune evasion, and recurrence. This section examines two cutting-edge approaches: optimizing Bacillus Calmette–Guérin (BCG)-induced immunity in bladder cancer and targeting protease-activated receptor 2 (PAR2) in ovarian cancer. Together, these strategies illustrate the transformative potential of immune modulation in revolutionizing cancer care [17].

BCG-induced anti-tumor immunity represents a significant milestone in bladder cancer therapy. The discovery of CXCL10- and MHC-II-expressing neutrophils as mediators of this response marks an important step forward. These neutrophils enhance CD8+ T-cell recruitment and activation, amplifying localized anti-tumor responses without triggering myeloid-derived suppressor cells (MDSCs), which often dampen immunity. Biomarkers like urinary CXCL10 levels and MHC-II neutrophil expression provide actionable tools for patient stratification, enabling the more precise application of BCG therapy. However, practical barriers persist. Systemic side effects and varying efficacy in resistant cases underscore the need for improved delivery systems. Innovations such as nanoparticles or liposomal formulations could enhance intravesical retention while minimizing adverse effects. Furthermore, combining BCG with immune checkpoint inhibitors, such as anti-PD-1/PD-L1 antibodies, offers a promising strategy for sustaining anti-tumor immunity in refractory cases. Longitudinal studies validating biomarkers and exploring these combination therapies will be essential to optimize this approach. Expanding the application of BCG-based immune modulation to other immune-responsive cancers could further broaden its clinical impact [17].

The identification of PAR2 as both a biomarker and therapeutic target represents another breakthrough, particularly in ovarian cancer treatment. PAR2, overexpressed in high-risk tissues such as the fallopian tubes of BRCA mutation carriers, drives oncogenic pathways including Akt and Etk/Bmx signaling. Pc(4-4), a cyclic peptide specifically designed to disrupt PAR2 signaling, has shown remarkable efficacy in preclinical models by significantly reducing tumor burden and metastatic potential while sparing normal tissues. Translating these findings into clinical practice requires biomarker-driven trials to stratify patients based on PAR2 expression. Combining Pc(4-4) with existing therapies, such as PARP inhibitors or immune checkpoint inhibitors, may enhance anti-tumor activity and overcome resistance mechanisms. Additionally, PAR2-targeting strategies hold potential for other malignancies, such as pancreatic and colorectal cancers, where similar signaling pathways drive progression. Broader targeting of GPCR pathways in these contexts could open new avenues for immune-based therapies in traditionally hard-to-treat cancers [18].

While these innovations highlight the transformative potential of immune modulation, challenges remain. Resistance mechanisms, off-target effects, and limited access to advanced therapies pose significant barriers. Future efforts must prioritize biomarker validation, optimize delivery systems, and address cost and accessibility to ensure these therapies reach diverse populations.

Immune modulation represents a pivotal shift toward personalized oncology, but its success depends on sustained research, interdisciplinary collaboration, and equitable healthcare policies. Addressing these challenges is essential to fully realize the promise of immune-based therapies and improve outcomes for patients worldwide.

## 5. Precision Oncology: Leveraging Molecular and Metabolic Insights

Precision oncology has reshaped cancer care by tailoring treatments to the unique molecular and metabolic profiles of individual tumors, offering a more personalized and effective approach. Emerging strategies, such as targeting metabolic vulnerabilities in KRAS-mutant pancreatic cancer and leveraging perioperative immune modulation with dexmedetomidine, exemplify the promise and challenges of this paradigm.

KRAS-mutant pancreatic cancer, one of the most aggressive malignancies, frequently reprograms its metabolism to sustain growth and evade therapy. Targeting TRPML1, a lysosomal ion channel critical for glutathione homeostasis and oxidative stress regulation, has shown promise in disrupting these pathways. Preclinical studies indicate that TRPML1 inhibition induces redox imbalance and selectively kills cancer cells, with biomarkers like taurine and 2-ketobutyric acid emerging as potential tools for monitoring therapeutic response. However, tumor cells exhibit remarkable adaptability to metabolic stress, often compensating through alternative pathways. To counter this, combination therapies targeting multiple metabolic nodes, such as glutaminase or lactate dehydrogenase, alongside immune checkpoint inhibitors, could provide more robust and sustained anti-tumor effects.

While metabolic targeting holds immense potential, challenges in clinical translation persist. Tumor heterogeneity, variability in biomarker expression, and inter-patient differences can hinder treatment efficacy. Standardizing biomarker testing protocols and incorporating complementary diagnostic tools, such as liquid biopsies, could enhance patient selection and improve outcomes. Future research must also identify additional metabolic vulnerabilities and refine therapeutic combinations to outpace tumor adaptability [19].

The perioperative period presents another critical opportunity to influence cancer outcomes. Dexmedetomidine, a selective alpha-2 adrenergic agonist, has demonstrated potential as an immunomodulatory agent during this window. Studies suggest that dexmedetomidine enhances anti-tumor immunity, reduces metastasis risk, and improves overall survival. By modulating the immune microenvironment, it creates an opportunity to synergize with other immunotherapies, such as checkpoint inhibitors or adoptive cell therapy. For instance, checkpoint inhibitors reinvigorate exhausted T cells, while dexmedetomidine modulates inflammation, improving T-cell activity and infiltration. Further research is needed to optimize dosing, understand the precise mechanisms, and identify patient populations that would benefit most from these combined approaches [20].

Despite these advances, precision oncology faces significant hurdles. Tumor resistance mechanisms, off-target effects, and the high variability of biomarker expression complicate the implementation of these therapies. Additionally, the cost and accessibility of advanced treatments remain pressing issues, particularly in resource-limited settings. Bridging the gap between innovation and widespread clinical application will require rigorous clinical trials, investment in scalable technologies, and policy reforms to ensure affordability and access.

The transformative potential of precision oncology lies in its ability to deliver targeted, effective therapies while minimizing toxicity. However, realizing this vision requires addressing critical knowledge gaps, fostering interdisciplinary collaboration, and prioritizing equitable access. As these innovative strategies evolve, their adoption must be guided by robust evidence, practical feasibility, and a commitment to improving outcomes for all patients. This moment represents a turning point in oncology, where scientific discovery must converge with clinical application to redefine cancer care. The urgency of this mission cannot be overstated—precision oncology is not just a scientific breakthrough; it is a moral imperative to improve the quality of life for those affected by cancer.

## 6. Conclusions

In conclusion, this Special Issue highlights the rapid advancements in cancer research and the transformative potential of emerging technologies. By understanding the underlying molecular and cellular mechanisms of cancer, researchers are developing innovative therapeutic strategies, diagnostic tools, and preventive measures. However, significant challenges remain, including overcoming drug resistance, minimizing side effects, and ensuring equitable access to advanced treatments.

As we continue to unravel the complexities of cancer, interdisciplinary collaboration and a patient-centered approach will be crucial. By fostering a global research community and prioritizing patient needs, we can accelerate the development of effective cancer treatments and improve the lives of countless individuals affected by this disease.

## References

[1] Sato H., Hara T., Meng S., Tsuji Y., Arao Y., Sasaki K., Miyoshi N., Kobayashi S., Doki Y., Eguchi H. (2023). Drug Discovery and Development of miRNA-Based Nucleotide Drugs for Gastrointestinal Cancer. Biomedicines.

[2] Meng W., He C., Hao Y., Wang L., Li L., Zhu G. (2020). Prospects and challenges of extracellular vesicle-based drug delivery system: Considering cell source. Drug Deliv..

[3] Watabe S., Aruga Y., Kato R., Kawade G., Kubo Y., Tatsuzawa A., Onishi I., Kinowaki Y., Ishibashi S., Ikeda M. (2023). Regulation of 4-HNE via SMARCA4 Is Associated with Worse Clinical Outcomes in Hepatocellular Carcinoma. Biomedicines.

[4] Raveendran S., Giram A., Elmi M., Ray S., Ireson C., Alavijeh M., Savina I.N. (2024). Combinatorial Therapy: Targeting CD133+ Glioma Stem-like Cells with a Polysaccharide–Prodrug Complex Functionalised Gold Nanocages. Biomedicines.

[5] Wu T., Pu C., Wang Q., Zhang K. (2023). Comparison of Efficacy and Safety of Anti-Programmed Cell Death-1 Antibody Plus Lenvatinib and Chemotherapy as First-Line Therapy for Patients with Stage IV Gallbladder Cancer: A Real-World Study in a Chinese Population. Biomedicines.

[6] Sung H., Ferlay J., Siegel R.L., Laversanne M., Soerjomataram I., Jemal A., Bray F. (2021). Global Cancer Statistics 2020: GLOBOCAN Estimates of Incidence and Mortality Worldwide for 36 Cancers in 185 Countries. CA Cancer J. Clin..

[7] Etzioni R., Urban N., Ramsey S., McIntosh M., Schwartz S., Reid B., Radich J., Anderson G., Hartwell L. (2003). The case for early detection. Nat. Rev. Cancer.

[8] Boyd N.F., Guo H., Martin L.J., Sun L., Stone J., Fishell E., Jong R.A., Hislop G., Chiarelli A., Minkin S. (2007). Mammographic density and the risk and detection of breast cancer. N. Engl. J. Med..

[9] Siegel R.L., Miller K.D., Fuchs H.E., Jemal A. (2022). Cancer statistics, 2022. CA Cancer J. Clin..

[10] Ostrom Q.T., Cioffi G., Waite K., Kruchko C., Barnholtz-Sloan J.S. (2021). CBTRUS Statistical Report: Primary Brain and Other Central Nervous System Tumors Diagnosed in the United States in 2014–2018. Neuro-Oncol..

[11] Ungkulpasvich U., Hatakeyama H., Hirotsu T., di Luccio E. (2023). Pancreatic Cancer and Detection Methods. Biomedicines.

[12] Pawlak M.E., Rudnicki W., Borkowska A., Skubisz K., Rydzyk R., Łuczyńska E. (2023). Comparative Analysis of Diagnostic Performance of Automatic Breast Ultrasound, Full-Field Digital Mammography and Contrast-Enhanced Mammography in Relation to Breast Composition. Biomedicines.

[13] Wilson M.L., Fleming K.A., Kuti M.A., Looi L.M., Lago N., Ru K. (2018). Access to pathology and laboratory medicine services: A crucial gap. Lancet Lond. Engl..

[14] Malone N.C., Williams M.M., Smith Fawzi M.C., Bennet J., Hill C., Katz J.N., Oriol N.E. (2020). Mobile health clinics in the United States. Int. J. Equity Health.

[15] Denny L., Kuhn L., De Souza M., Pollack A.E., Dupree W., Wright T.C. (2005). Screen-and-treat approaches for cervical cancer prevention in low-resource settings: A randomized controlled trial. JAMA.

[16] Esteva A., Robicquet A., Ramsundar B., Kuleshov V., DePristo M., Chou K., Cui C., Corrado G., Thrun S., Dean J. (2019). A guide to deep learning in healthcare. Nat. Med..

[17] Takeda Y., Kato T., Sabrina S., Naito S., Ito H., Emi N., Kuboki Y., Takai Y., Fukuhara H., Ushijima M. (2023). Intracellular Major Histocompatibility Complex Class II and C-X-C Motif Chemokine Ligand 10-Expressing Neutrophils Indicate the State of Anti-Tumor Activity Induced by Bacillus Calmette-Guérin. Biomedicines.

[18] Nag J.K., Grisaru-Granovsky S., Armon S., Rudina T., Appasamy P., Bar-Shavit R. (2024). Involvement of Protease-Activated Receptor2 Pleckstrin Homology Binding Domain in Ovarian Cancer: Expression in Fallopian Tubes and Drug Design. Biomedicines.

[19] Kim B., Jung J. (2024). Metabolomic Approach to Identify Potential Biomarkers in KRAS-Mutant Pancreatic Cancer Cells. Biomedicines.

[20] Moon J., Chun D.-H., Kong H.J., Lee H.S., Jeon S., Park J., Kim N.Y., Kim H.-I. (2023). The Intraoperative Administration of Dexmedetomidine Alleviates Postoperative Inflammatory Response in Patients Undergoing Laparoscopy-Assisted Gastrectomy: A Double-Blind Randomized Controlled Trial. Biomedicines.

